# Antioxidant and Anti–Aging Activity of Freeze–Dried Alcohol–Water Extracts from Common Nettle (*Urtica dioica* L.) and Peppermint (*Mentha piperita* L.) in Elastomer Vulcanizates

**DOI:** 10.3390/polym14071460

**Published:** 2022-04-03

**Authors:** Marcin Masłowski, Andrii Aleksieiev, Justyna Miedzianowska, Magdalena Efenberger-Szmechtyk, Krzysztof Strzelec

**Affiliations:** 1 Institute of Polymer & Dye Technology, Lodz University of Technology, Stefanowskiego 16, 90-537 Lodz, Poland; andrii.aleksieiev@dokt.p.lodz.pl (A.A.); justyna.miedzianowska@p.lodz.pl (J.M.); krzysztof.strzelec@p.lodz.pl (K.S.); 2 Institute of Fermentation Technology & Microbiology, Lodz University of Technology, Wolczanska 171/173, 90-530 Lodz, Poland; magdalena.efenberger-szmechtyk@p.lodz.pl

**Keywords:** common nettle, peppermint, natural rubber, freeze–dried extracts, antioxidants, bio–additives, elastomer vulcanizates

## Abstract

The research article aimed to examine the antioxidant nature of freeze–dried extracts from *Urtica dioica* L. and *Mentha piperita* L. and to present a deep characterization of their influence on the properties of natural rubber–based vulcanizates before and after simulated aging processes. Natural extracts were prepared in three solvent systems at selected volume ratios: water (100), methanol–water (50/50), ethanol–water (50/50), which were further lyophilized and used as additive to natural rubber mixtures. Freeze–dried materials were investigated by UV–VIS diffuse reflectance spectroscopy, Fourier Transform Infrared Spectroscopy (FTIR), thermal stability by thermogravimetric analysis (TGA). Antioxidant activity and total phenolic content (TPC) were also examined. Prepared samples were subjected to accelerated simulated degradation processes by using ultraviolet and thermo-oxidative aging. Vulcanizates resistance to degradation effects was determined by the study of cross-linking density (equilibrium swelling method), mechanical properties (tensile strength, elongation at break) and color change in comparison with the results of the reference samples. The research showed that analyzed extracts are characterized by a high content of polyphenols and antioxidant activity, thus have a protective influence on elastomer vulcanizates against damaging effects of aging processes, which consequently extends the lifetime of materials.

## 1. Introduction

It is common knowledge that over centuries plants have been used for medicinal purposes [1,2,3], cosmetics [4,5] and as a food component [6,7] all over the world due to various biological activities. Plants used for such applications belong to the group of herbs. The main reason for the favourable properties of herbs is the presence of phytochemicals, including secondary metabolites [8]. These organic compounds are produced in higher plants and most cases are not necessarily needed for growth and development but often occur as a response to biotic and abiotic external factors [9]. The majority of secondary metabolites in herbs are terpenoids, alkaloids and flavonoids, which are substances scientifically confirmed as an antioxidant, antibacterial, anti-inflammatory, anti-aging and antitumor [10,11,12,13]. Among the most commonly known antioxidants, the following substances can be mentioned: luteolin, quercetin, hesperetin (polyphenols); lutein, β-carotene (carotenoids); lycopene, resveratrol, hydroxytyrosol (phenols) and vitamins A, C, E. Depending on the needs, herbs are applied as freshly harvested or dried as well as processed in the form of essential oils or extracts. 

Today’s technological development allows to obtain plant extracts with higher efficiency using advanced methods, but often these processes are associated with higher costs due to the necessary equipment, the use of an additional pressure source, increased temperature, electricity consumption and others [14,15,16]. The most commonly known and cheap tactic is the extraction prepared in aqueous, alcoholic or other liquid solutions by various methods, using the whole plant or its parts (flowers, leaves, roots, fruits) [17]. Many works associated with selected parts of the herb or plant aimed to determine the content of specific compounds responsible for required biological activity [18,19,20]. Studies on natural extracts from many plants have proven their antioxidant, antimicrobial, antifungal, antibacterial, anti-inflammatory, antipyretic and analgesic activity and, as a consequence, wide potential application [21,22,23,24,25,26,27,28]. Among many plants with various biological effects, *Mentha piperita* L. and *Urtica dioica* L. are species with intensive antioxidant and anti-aging properties associated with the presence of various phytochemicals, such as terpenoids, carotenoids, tannins, vitamins, acids and many derivatives [29,30,31]. Bourgeois et al. confirmed high content of ursolic acid, quercetin and other phenolic compounds in nettle extracts [32], while Pavlić et al. [33] provided information about the composition of peppermint extracts, which included terpenes and its derivatives (menthol, menthone, iso-menthol, eucalyptol, iso-menthone) and other lipophilic bioactives.

*Urtica dioica* L. (also known as stinging nettle or common nettle) is herbaceous plant, which belongs to the Urticaceae family in the major group of Angiosperms (flowering plants) [34]. Vernacular name of the plant is related to its most characteristic feature, which is a presence of stinging hairs. Common nettle is a erect perennial plant that grows up to two metres tall, it has opposite ovate-lanceolate leaves, toothed margins with acute tip, axillary inflorescence with many small, green and unisexual flowers, and achenes as its fruits [35]. It is known for its rapid growth especially in shady humid places mainly forests in temperate climate zones throughout Europe, Asia, North Africa and North America [36]. Due to the wide availability of common nettle in regions presented above, it can be harvested from natural habitats or from cultivation areas. According to studies, *Urtica dioica* L. is rich in different amount of ingredients depending on the part of the plant and the region of habitat. Aerial parts are rich in proteins, terpenoids, carotenoids, lutein and lycopene, fatty acids, polyphenolic compounds, essential amino acids, chlorophyll, vitamins (A, C, D, E, F, K and P), tannins, carbohydrates, sterols, polysaccharides, isolectins, minerals and metals such as iron, selenium, zinc, calcium and magnesium [34]. Major secondary metabolites, including antioxidants, provide the antioxidant, antifungal, anti-inflammatory [37] and anti-aging [38] activity of the plant. Antioxidants protect cells against the oxidative stress associated with the action of free radicals, which are considered to be the primary cause of aging processes [39]. The use of common nettle shown beneficial therapeutic effects for various ailments predominantly as a blood nourishing tonic and for seasonal rhinitis [40,41,42] but also toward urinary ailments, specifically with the urinary tract and kidney stones [43]. 

*Mentha piperita* L. (commonly known as peppermint) is a natural hybrid of *Mentha aquatica* L. and *Mentha spicata* L. and belongs to the Lamiaceae Family [44]. It is a herbaceous rhizomatous perennial plant, which grows up to one meter tall, it has smooth stems, dark green leaves with an acute apex and coarsely toothedmargins, wide-spreading rhizomes and bare fibrous roots [39]. The flowers are purple with a four-lobed corolla. The plant grows wild and spreads quickly in moist habitats, primarily in Europe, North America and Asia, but it is cultivated worldwide [45]. Mints are characteristic for their aromatic odour and warm burning taste followed by the feeling of coolness, which is related to the high content of menthol [46]. Besides menthol, peppermint consists of menthone, menthyl acetate, carvone, linalool, limonene, pinene and various flavonoids such as eriocitrin, narirutin, hesperidin, isorhoifolin, diosmin, rosmarinic acid and others [45,47]. Singh et al. proved the antibacterial and antioxidant properties of peppermint oil and extract, which inhibited the process of microbial growth [48], while Sarikhani et al. examined the anti-aging properties of the peppermint extract [49]. High antioxidant and anti-aging activity is probably related to the presence of phenolic components.

Besides major applications of plants as substances for healing and relieving symptoms of various diseases by oral ingestion or the external application as essential oils [50,51] and extracts [52], successful attempts have been made to use antioxidant and anti-aging abilities of selected plants to improve the properties of elastomer composites [53,54,55,56,57].

Following the increase of ecological consciousness and environmental problems corresponding to the reduction of non-renewable materials, many researchers focused on the new natural solutions in the field of polymer technology [58,59,60,61,62,63]. The purpose of conducted studies is the partial or complete replacement of synthetic polymers, rubbers and additives with renewable raw materials. The most commonly known example of the naturally existing high-molecular and non-toxic elastomer is natural rubber (NR). It is obtained in the form of latex or milky colloidal suspension from tropical trees and plants mainly from *Hevea brasiliensis* [64]. Due to its low cost and simultaneous above-average properties such as good mechanical strength, electrical insulation, water resistance, most salts and alkalis, natural rubber is widely used elastomer in various fields of industry [65]. It found its application in textiles, belts, coated fabrics, insulations, cables, bands, tires and many more [66,67,68,69,70]. To improve the technical and exploitation properties of NR-based vulcanizates such as tensile strength, barrier properties or thermal conductivities, natural or synthetic fillers and additives are often used [55,71,72,73]. Following the general trend to reduce the usage of synthetic components in polymer technology by replacing them with renewable raw materials, the authors of the manuscript aimed to investigate the influence of antioxidant freeze-dried natural extracts on the elastomer mixture’s resistance to simulated aging processes. To obtained vulcanizates based on the natural rubber, freeze-dried extracts from *Mentha piperita* L. and *Urtica dioica* L. were added. Materials were subjected to accelerated simulated thermo-oxidative and UV aging and then aged and non-aged samples were studied. Moreover, the characterization of obtained freeze-dried extracts was carried out to provide an in-depth analysis of their antioxidant and anti-aging properties and potential application as bio–additives in elastomer vulcanizates. The article is inspired by the previous research studies on the NR vulcanizates with the addition of freeze-dried *Equisteum arvense* extract [53] and is presented to extend the knowledge on the subject of natural additives used in elastomer technology.

## 2. Materials and Methods

### 2.1. Materials

- Elastomer matrix: natural rubber RSS I (NR) provided by Torimex Chemicals Sp. z o.o. (Konstantynów Łódzki, Poland).
- Cross-linking system: sulphur (Siarkopol, Tarnobrzeg, Poland), stearin (POCH, Gliwice, Poland), 2-mercaptobenzothiazole (MBT) (Sigma-Aldrich, Poznań, Poland), micro-sized zinc oxide (ZnO) (Huta Będzin, Będzin, Poland).
- Dried plants: *Mentha piperita* L. (MP) provided by ManuTea (Chałupki, Poland) and *Urtica dioica* L. (CN) provided by Ziołowy Zakątek (Koryciny, Poland).

Both plants used for this study was purchased from certificated food companies. Leaves of plants were harvested in Podlasie (Poland) before the flowering: peppermint in June, common nettle between April and May. Dried leaves were packed into paper bags. Products were ground in food chopper to obtain powders.

### 2.2. Preparation of Freeze-Dried Extracts

Three different solvents were prepared to perform extraction: pure water (W); methanol–water (W/M) and ethanol–water (W/E) in 50/50 volume. The 15 g of dried and ground plant material was placed in a cellulose thimble and immersed in a selected solvent (100 mL). Performed extraction was a three-stage process conducted in a Series 148 extractor from Velp (Usmate Velate, Italy). Prepared mixture of solvent and plant material was brought to a boiling temperature and the extraction process was carried out for 1.5 h. Afterward, the thimbles were lifted over the solvent and rinsed with the evaporating solvent (step 1). Then, another 15 g portion of plant material was added to the new thimble and beaker with obtained extract were filled with solvent to the volume of 100 mL. The thimbles were immersed again for another 1.5 h from the reflux point. Further steps were identical to step 1 (step 2). In step 3, the thimble was changed for the last time. After the extraction, the solvent was evaporated in rotary evaporator Heidolph, Laborata 4001 (Teltow, Germany) to the volume of ca. 40 mL.

To obtain products in solid form, extracts were subjected to the freeze–drying process in Labconco Freezzone 2.5 Plus (Kansas City, MN, USA). 

### 2.3. Preparation of Rubber Mixtures and Vulcanizates

Elastomer mixtures were obtained in the following steps:

- Plastification of natural rubber in Brabender measuring mixer N50 (Brabender Technologie GmBH & Co. KG, Duisburg, Germany) for 4 min with a rotational speed of 40 rpm and a temperature range of 40–60 °C.
- Addition of freeze-dried extracts to the plasticized natural rubber under the same conditions.
- Mixing with the cross–linking system using a two–roll mill at room temperature.
- Preparation of separate mixture without freeze-dried extracts as a reference sample.

The composition of prepared rubber mixtures are presented in Table 1.

For the vulcanization process steel molds containing rubber mixtures were placed between the shelves of an electrically heated hydraulic press. Prepared mixtures were vulcanized at the temperature of 160 °C and at 15 MPa pressure for curing time determined from rheometric measurements.

### 2.4. Methods Used for Freeze-Dried Extracts

#### 2.4.1. Fourier Transform Infrared Spectroscopy (FTIR)

FTIR spectra were recorded for freeze-dried extracts using Nicolet 6700 spectrophotometer (Thermo Fischer Scientific Instruments, Waltham, MA, USA) equipped with a diamond adapter (Smart Orbit ATR sampling accessory—(Thermo Fischer Scientific Instruments, Waltham, MA, USA)). The analysis was performed with 128 scans over the range of 3800–800 cm^−1^.

#### 2.4.2. UV–VIS Diffuse Reflectance Spectroscopy

The Evolution 201/220 UV–Visible Spectrophotometer (Thermo Scientific, Waltham, MA, USA) equipped with was used to obtain UV–VIS spectra of prepared freeze-dried extracts. The device was equipped with xenon, tungsten and deuterium lamps as a source of light. Materials were placed in the glass cuvettes. Measurements of the natural extracts were conducted in the spectral range of 1100 to 200 nm in the “Scan” mode (measurement of the light passing through the sample over the entire spectral range).

#### 2.4.3. Near–Infrared Spectroscopy (NIR)

To achieve analysis of bonds characteristic to freeze-dried extracts structure in the electromagnetic spectrum of 10,000–4000 cm^−1^ range, the Nicolet 6700 spectrophotometer (Thermo Fischer Scientific Instruments, Waltham, MA, USA) was used. Spectra were recorded in absorption mode—128 scans and 8 cm^−1^ resolution.

#### 2.4.4. Thermogravimetric Analysis (TGA)

The thermal stability of freeze-dried extracts were tested using the TGA/DSC1 analyzer (Mettler Toledo, Columbus, OH, USA/Greifensee, Switzerland). The analysis was conducted in the temperature range of 25 to 600 °C with a 10 °C/min heating rate in a flow of nitrogen at 60 mL/min.

#### 2.4.5. Total Phenolic Content (TPC)

The total phenolic content (TPC) of the extracts was determined using the Folin–Ciocalteu method described by Singleton and Rossi (1965). The reaction mixture consisted of: 100 µL of the extract; 200 µL of Folin–Ciocalteu reagent; 1 mL of 20% Na_2_CO_3_ solution; and 2 mL of distilled water. The blank sample contained 100 µL of distilled water instead of the extract. The samples were mixed and stored at room temperature in a dark place for 1 h. The absorbance was measured at the wavelength of 765 nm against the blank sample using a Cecil CE2041 spectrophotometer (Cecil Instruments Limited, Cambridge, UK). A calibration curve for gallic acid was prepared. The TPC was quantified according to a calibration curve and expressed as gallic acid equivalents (µgGAE/mL).

#### 2.4.6. Antioxidant Activity

Antioxidant activity of the extracts was measured with two free radical scavenging methods: DPPH and ABTS as it was previously described by Efenberger-Szmechtyk et al. [74].

- DPPH

2.4 mg of 2,2-diphenyl-1-picrylhydrazyl (DPPH) (Sigma-Aldrich, St. Louis, MO, USA) was dissolved with 100 mL of 80% ethanol. Then, 50 µL of plant extract was added to 1.95 mL of the DPPH free radical solution. The samples were kept for 15 min at room temperature in a dark place. The absorbance was measured at the wavelength of 515 nm against 80% ethanol, using a Cecil CE2041 spectrophotometer (Cecil Instruments Limited, Cambridge, UK). The antioxidant activity was quantified according to the calibration curve prepared for Trolox (6-hydroxy-2,5,7,8-tetramethylchroman-2-carboxylic acid) (Sigma-Aldrich, St. Louis, MO, USA) and expressed as the concentration of Trolox equivalents (mgTE/mL).

- ABTS

ABTS (2,2-azinobis(3-ethylbenzothiazoline-6-sulphonic acid) (Sigma-Aldrich, St. Louis, MO, USA) was dissolved in water to obtain a concentration equal to 7 mmol. K_2_S_2_O_8_ (final concentration of 2.45 mmol) was added to ABTS stock solution and stored for 12–16 h in a dark place at room temperature to obtain ABTS radical cation (ABTS+). The ABTS+ solution was diluted to reach an absorbance of 0.700 measured at 734 nm. The reaction mixtures contained 3 mL of diluted ABTS+ solution and 30 µL of extract. The absorbance was measured after 10 min at the wavelength of 734 nm against distilled water using a Cecil CE2041 spectrophotometer (Cecil Instruments Limited, Cambridge, Great Britain). The antioxidant activity was calculated based on the calibration curve prepared for Trolox and expressed as the concentration of Trolox equivalents (mgTE/mL).

Mean values and standard deviations (SD) were calculated. Statistically significant differences between samples were calculated with one-way ANOVA with Tukey’s honestly significant differences (HSD) test (*p* < 0.05) using R 3.4.0 (R Core Team, Vienna, Austria).

### 2.5. Methods Used for Elastomer Vulcanizates

#### 2.5.1. Rheometric Properties

To determine the rheometric properties of rubber mixtures, samples were placed in the measuring chamber of the MonTech DRPA 300 Rheometer (MonTech Werkstoffprüfmaschinen GmbH, Buchen, Germany) and subjected to changes in the torque of the oscillating disc as a function of time in 160 °C. During tests, two parameters were measured: optimal curing time (t_90_) and increase in torque (ΔM). Merck (1000 mg/L) standard solutions were prepared for calibration curves.

#### 2.5.2. Aging Processes

Vulcanizates were subjected to the accelerated simulation of thermo–oxidative and ultraviolet aging. Thermo–oxidative simulation was performed in a forced air dryer Binder Model FED 56 (BINDER GmbH, Tuttlingen, Germany) at 70 °C for 14 days. Ultraviolet process was carried out in the UV chamber of Atlas UV 2000 (ATLAS Material Testing Technology GmbH, Duisburg, Germany). The UV chamber was set in the following conditions: the day and night segment: 0.78 W/m^2^; temperature: 60 °C; duration: 72 h.

#### 2.5.3. Cross–Linking Density

Non–aged and aged vulcanizates were tested for the cross–linking density of the spatial network. Examinations were conducted according to the solvent–swelling measurements in toluene. Results were calculated from the Flory–Rehner Equation (1) [75]:

(1) $$\gamma_{e}=\frac{\ln\left(1-V_{r}\right){+V}_{r}+\mu V_{E}^{2}}{V_{0}\left(V_{r}^{\frac{1}{3}}-\frac{V_{r}}{2}\right)}$$

where: $\gamma_{e}$—the cross-linking density (mol/cm^3^), V_0_—the molecular volume of solvent (106.7 cm^3^/mol), μ—the Huggins parameter of the NR-solvent interaction calculated from Equation (2):

(2) $${\mu =\mu }_{0}+\beta \cdot V_{r},$$

where: μ_0_—the parameter connected with non-cross-linked/solvent, β—the constant consideration of the impact of cross-linking on parameter polymer/solvent, natural rubber–toluene interaction factor μ_0_ and β were experimentally (μ_0_ = 0.478, β = 0.228); V_r_—the volume fraction of elastomer in the swollen Equation (3):

(3) $$V_{r}=\frac{1}{{1+Q}_{w}\frac{\rho_{k}}{\rho_{r}}},$$

where: Q_w_—weight of equilibrium swelling, Q_k_—density of rubber (g/cm^3^) (0.99 g/cm^3^), Q_r_—density of solvent (g/cm^3^) (0.86 g/cm^3^).

#### 2.5.4. Barrier Properties

To investigate the vulcanizates barrier properties, the manometric method was used. Tests were performed according to the ASTM standard D1434 based od through–plane air permeability and the gas transmission rate (GTR) was calculated from the following Equation (4) [76]:

(4) $$\mathrm{GTR}=\frac{V_{c}}{R\cdot T\cdot P_{u}\cdot A}\cdot \frac{\mathrm{dp}}{\mathrm{dt}},$$

where: V_c_—the volume of the low-pressure chamber (L), T—temperature (K), P_u_—the gas pressure in the high-pressure chamber (Pa), A—area permeation of gas through the sample (m^2^), dp/dt—pressure changes per unit time (Pa/s), R—gas constant 8.31 × 10^3^ ((L·Pa)/(K·mol)).

#### 2.5.5. Mechanical Properties

Dumbbell–shaped samples of aged and non–aged vulcanizates were examined according to ISO-37 using a static material testing machine Zwick (model 1435, Ulm, Germany). Tests were conducted at room temperature and at the cross–head speed of 500 mm/min. Measurements were performed to determine tensile strength (TS), elongation at break (E_b_) and aging coefficient (K) according to the following Equation (5) [77]:

(5) $$K=\frac{{(\mathrm{TS}\cdot E_{b})}_{\mathrm{after}\;\mathrm{aging}}}{{(\mathrm{TS}\cdot E_{b})}_{\mathrm{before}\;\mathrm{aging}}},$$

#### 2.5.6. Color Stability

Aged and non–aged elastomer vulcanizates were tested according to the PN-EN ISO 105-J01 standard using the Konica Minolta CM-3600d spectrophotometer (Sony, Tokyo, Japan) to analyze the influence of degradation factors on color stability of samples. The measurements were conducted in the spectral range of 360–740 nm. The total color change was determined according to the CIE-Lab color space from Equation (6) [78]:

(6) $${\mathrm{dE}}_{\mathrm{ab}}^{*}=\sqrt{\Delta a^{2}+\Delta b^{2}+\Delta L^{2}},\;$$

where: Δa—deviation from the color of the reference sample in the axis of red—green; Δb—deviation from the color of the reference sample in the axis of yellow—blue, ΔL—deviation in brightness parameter from the color of the reference sample.

## 3. Results

### 3.1. Characteristic of Freeze-Dried Extracts

#### 3.1.1. Fourier Transform Infrared Spectroscopy (FTIR)

**Figure 1:**
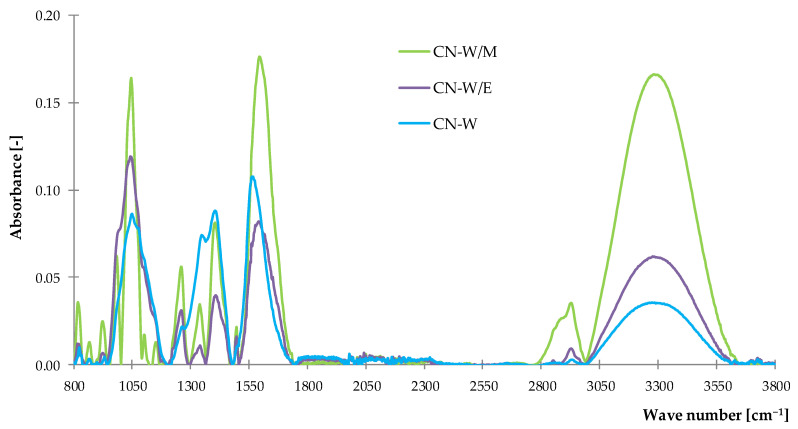
The FTIR spectra of common nettle freeze-dried extracts.

**Figure 2:**
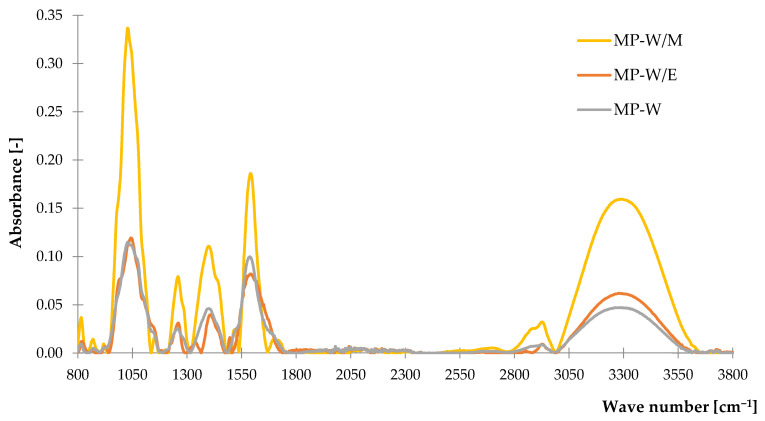
The FTIR spectra of peppermint freeze-dried extracts.

Obtained FTIR spectra were arranged into two separate graphs to present results recorded for common nettle (Figure 1) and peppermint (Figure 2) freeze-dried extracts. In the case of both samples extracted in water-methanol solution, the highest intensity of recorded peaks was noticed with maximum values at 1050, 1600 and 3300 cm^−1^. Absorbance recorded at 1050 cm^−1^ is a characteristic peak for pure methanol spectrum according to the results presented by Doroshenko et al. [79]. This corresponds mainly to the stretching vibrations of the carbon-hydrogen bond and oxygen-hydrogen group. Besides it refers to the vibration of (–C–O) groups in ether, esters and carboxylic acids from tannins, flavonoids and other metabolites and it is characteristic for all of the investigated extracts. Groups registered at 1280 cm^−1^ are related to the presence of aliphatic and carbonyl stretching vibrations. Peaks observed in the range of 1300–1500 cm^−1^ are characteristic for several different groups listed in detail in Table 2. The (C=C) bond registered in this region is related to the aromatic rings, which indicate the presence of polyphenols in examined samples. Band at the 1390–1310 cm^−1^ of the spectrum is associated with the O–H bending vibrations of phenol groups. Stretching and deformation vibrations of methyl and methylene groups were recorded at 1600 cm^−1^, while asymmetric and symmetric vibrations of these groups were registered as a band in the range of 2800–2970 cm^−1^. Broad band noticed at 3000–3600 cm^−1^ with the maximum at 3300 cm^−1^ is related to the stretching vibrations of O–H bond of phenols and alcohols. The most intensive peaks in the range of 2800–3000 cm^−1^ were noticed for peppermint extract from ethanol-water (Figure 2).

#### 3.1.2. Near–Infrared Spectroscopy (NIR)

**Figure 3:**
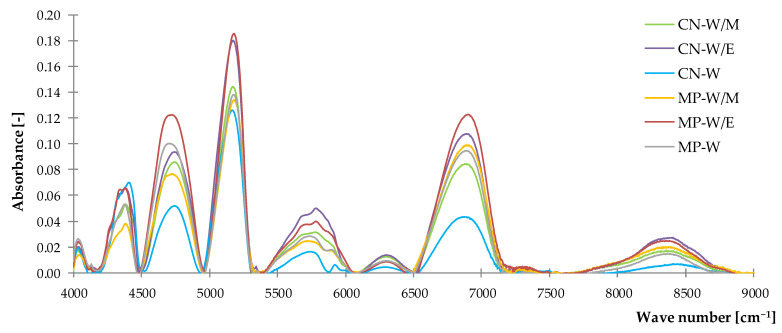
The NIR spectra of freeze-dried extracts.

The method of near-infrared spectroscopy was used to obtain an extended spectroscopic analysis of prepared extracts components. The results of conducted studies were analyzed by the assignment of peaks (Figure 3) to the presence of characteristic groups (Table 3). 

According to the literature [81], recorded spectra presented the high content of organic compounds in tested samples. Stretching vibrations from the C–H bond of these substances were noticed in several peaks in the range of 4034–4040 cm^−1^, 4375–4408 cm^−1^, 5551–5928 cm^−1^ (first overtone) and 8093–8550 cm^−1^ (second overtone). Registered vibrations may be related to the phenolic compounds [82,83] and carotenoids [84,85,86] as it was established by the FTIR spectra. The content of water was observed by the presence of O–H groups in several bands. 

According to the obtained spectra, the highest absorbance values were recorded for the freeze-dried extracts from ethanol-water solvents, while the water extracts presented decreased peak intensities. This tendency was observed for both common nettle and peppermint. It can be concluded that the content of determined substances differs in tested samples, which was proved by the FTIR analysis.

#### 3.1.3. UV–VIS Diffuse Reflectance Spectroscopy

**Figure 4:**
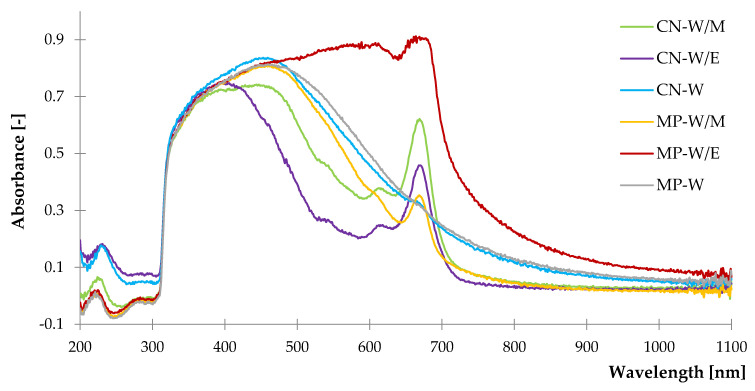
The UV-VIS spectra of freeze-dried extracts.

The analysis of the UV–VIS spectra (Figure 4) led to the identification of chromophores contained in the investigated freeze-dried extracts. The low intensive peak at ca. 220 nm registered in every sample corresponded to the unsaturated groups of ketones (e.g., menthone) and phenols [90]. According to Ernawati et al. [91], a noticeable increase at the wavelength of 300–350 nm is related to the occurrence of flavonoids, phenolic acids and derivatives. Besides, absorption ranges of 380–480 nm and 560–680 nm with characteristic peaks at 380 and 680 indicated the presence of chlorophyll “a” and “b” [92]. Moreover, all samples showed intense radiation absorption bands from 400 to 500 nm due to the content of carotenoids present in plant materials [93]. A slight decrease of spectra at 680 nm was noticed in the case of freeze-dried extracts from water due to the insolubility of chlorophyll in this particular solvent. The increased absorbance over 500 nm was noticed for the peppermint extracted from ethanol-water solution. Identified functional groups are related to the presence of secondary metabolites, which are concerned as antioxidant substances. It can be concluded that they may provide additional resistance to the aging processes, when added to natural rubber.

#### 3.1.4. Thermal Stability

**Figure 5:**
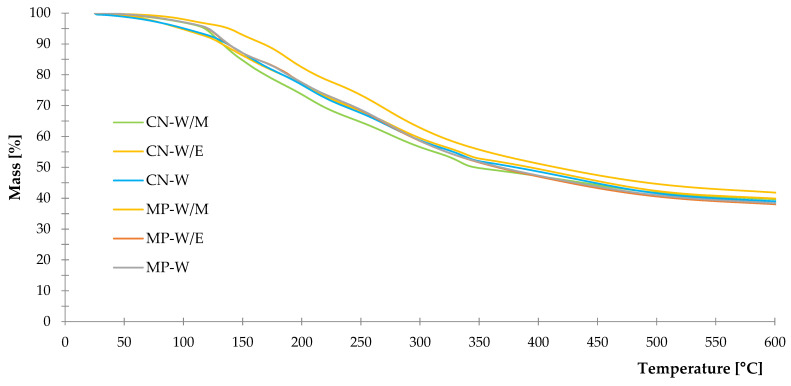
TG curves of freeze-dried extracts.

**Figure 6:**
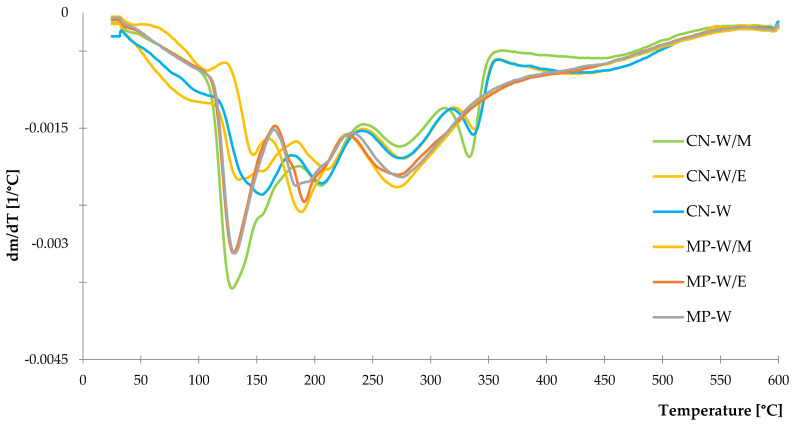
DTG curves of freeze-dried extracts.

Freeze-dried extracts were subjected to the examination of thermal stability by thermogravimetric analysis to obtain TG and DTG curves from room temperature to 600 °C. By analyzing the graph with results of TG measurements (Figure 5), it can be stated that the highest mass loss of all samples occurred in the range of 120–500 °C leaving the total residue at the temperature of 600 °C with ca. 35–40% of starting weight. A high percentage of remained mass was observed due to the non–oxidizing atmosphere used for the study. Detailed presentation of thermal degradation with the increasing temperature was obtained by the DTG relation (Figure 6), where it can be observed that thermal decomposition proceeds in a few stages. The first stage occurred in the range of 25–150 °C, which is directly related to the evaporation of remaining moisture and residues of solvents used for extraction. Rapid mass loss from 110 to 130 °C was recorded for three extracts: CN-W/M, MP-W/E and MP-W. It was noticed that the freeze-dried peppermint extract from methanol-water solution presented the lowest mass loss out of the rest samples in the described temperature range. The second stage was registered from 150 to 350 °C with several peaks related to the presence of secondary metabolites such as phenolic compounds, flavonoids and others [94,95,96]. The last stage over 350 °C was the utter degradation of samples. Percentage mass losses in individual temperature ranges are presented in Table 4. 

#### 3.1.5. Total Phenolic Content (TPC) and Antioxidant Activity

In this study, the extraction efficiency of phenolic compounds using different types of solvents (W/M, W/E, W) was examined. According to the results presented in Table 5. generally, *Mentha piperita* L. extracts showed significantly higher TPC values than *Urtica dioica* L. extracts. Antioxidant activity measured with two free radical scavenging methods was high and correlated with TPC. In terms of peppermint, the highest TPC was detected in methanolic extract followed by ethanolic and water extracts. As far as common nettle extracts are concerned, the most phenolic compounds were detected in ethanolic extract and the least contained water extract. In addition, for both plants significantly lower TPC values were observed in water extracts when compared to solutions obtained with organic solvents. These differences can be due to the different solubility of polyphenols in organic and inorganic solvents. Literature data suggest that a mixture of solvents gives the highest extraction efficiency [97,98].

In our previous studies, we extracted bioactive compounds from horsetail (*Equisteum arvense* L.) [53], but in this study, we managed to obtain extracts with significantly higher TPC and antioxidant activity. Probably, peppermint and common nettle contain more polyphenols than horsetail.

According to literature, *Mentha piperita* L. is a great source of polyphenols [99]. Khanzada et al. [100] reported that peppermint contained significantly higher amounts of phenolic compounds compared to other plants. It contains phenolic acids including hydroxybenzoic acids (protocatechuic, vanillic) and hydroxycinnamic acids (chlorogeic acid derivatives, rosmarinic, caffeic, p-coumaric, sinapic, caftaric, fertaric, and coutaric acid) as well as flavonoids: flavonols (kaempferol, quercetin, isorhamnetin derivatives), flavones (luteolin derivatives), flavanones (naringenin derivatives, hesperidin) and flavan-3-ols (epicatechin). Lignans and stilbenes were also found [99,101]. Moreover, it was shown that rosmarinic acid is one of the predominant compounds found in *Mentha piperita* L. leaves [99,102].

In the *Urtica dioica* L. leaves, the following phenolic acids were identified: benzoic acids (protocatechuic, genistic, syringic, gallic) and cinnamic acids (caffeic, chlorogenic, p-coumaric, cinnamic, ferulic, sinapic, chicoric acid). It also contained flavonoids belonging to flavonols (quercetin, isorhamnetin kaempferol derivatives and myricetin) flavan-3-ols (epigallocatechin gallate, epicatechin, catechin, epicatechin gallate), flavones (luteolin, apigenin, apigenin hexoside), isoflavones (genistein), flavanones (naringenin) [103,104]. Coumarins such as umbelliferone, esculetin and scopoletin [104] and carotenoids were also detected.

### 3.2. Characteristic of Vulcanizates

#### 3.2.1. Rheometric Properties

Rubber mixtures were subjected to the rheometric measurements to define the optimal vulcanization time (t_90_) and increase in torque (ΔM). The t_90_ parameter is the time required for the torque to reach 90% of the maximum value [105], while the ΔM parameter is the increase in torque measured as a difference of maximum and minimum torque registered during the test [106].

**Figure 7:**
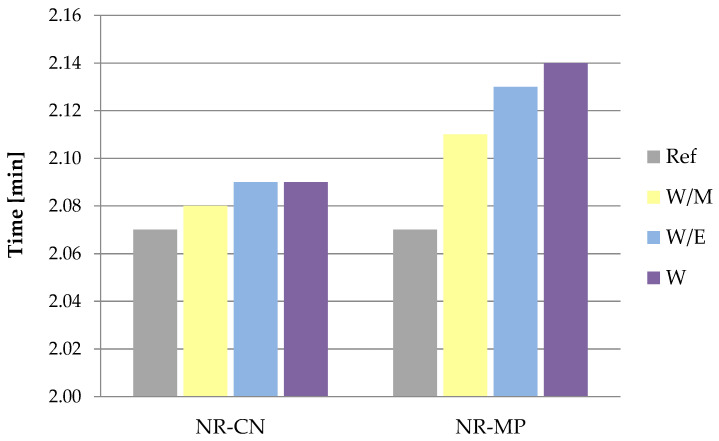
Optimal vulcanization time (t_90_) of vulcanizates.

**Figure 8:**
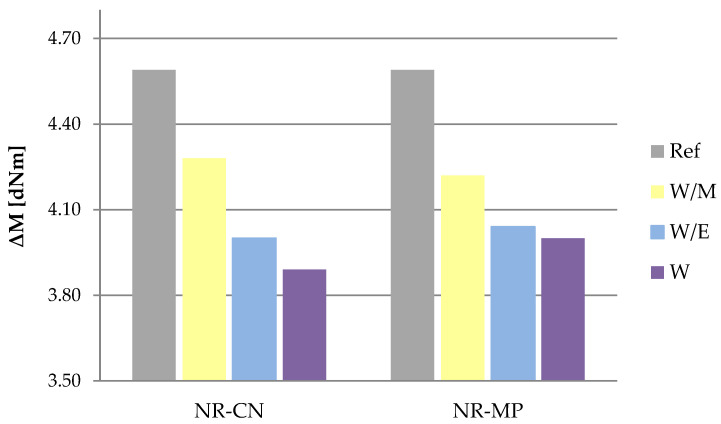
Increase in torque (ΔM).

According to the results (Figure 7), the optimal vulcanization time of vulcanizates containing bio–additives slightly extended in all samples. A higher increase was noticed in the case of vulcanizates with the addition of peppermint as the t_90_ values from 2.11 to 2.14 min, while the optimal vulcanization of the reference sample gained 2.07 min. These slight increases could be a result of the addition of the organic additive phase, which was mechanically introduced into the rubber mixtures and which may have caused the decrease of effectiveness in the activation and action of the curing system compounds. This is confirmed by the results of ΔM (Figure 8), which indicates the lower increase in torque in samples containing freeze-dried extracts. As the increase in torque is an indirect measurement of cross–linking density of vulcanizates, it can be assumed that the spatial network in reference vulcanizates would be more extensive than in vulcanizates containing freeze-dried extracts.

#### 3.2.2. Cross–Linking Density

During the aging process, elastomer vulcanizates are subjected to the influence of various aging factors and as a consequence vulcanizates may undergo degradation reactions or further cross–linking, so-called post–curing [107]. This occurs due to the recombination of free radicals into branched structures that expand the internal spatial network of the polymer. Another possibility is the degradation of natural rubber, which is subjected to uncontrolled and irreversible changes in polymer structure such as chain scissions and the formation of chain molecules, which leads to the reduction of elastomer molecular weight. These entangled polymer chains reduce the mobility of the molecules and thus the flexibility of the vulcanizate, leading to decreased swelling and increased cross–linking density (ν_e_). Elevated temperature, on the other hand, may lead to the activation of the remaining cross–linking system compounds used in the mixture and also contribute to the formation of new network nodes. As the cross–linking density of polymers is related to the aging factors, the ν_e_ parameters were presented for non-aged and aged samples (Table 6).

Among all vulcanizates, the highest cross–linking density was noticed for reference samples both unaged and aged, which correlate with the ΔM parameter defined by the rheometric measurements. The addition of freeze-dried extracts to the rubber mixtures caused a deterioration of ν_e_ parameter before simulated degradation processes compared to reference sample. On the other hand, a slight increase of cross–linking density was noticed for vulcanizates containing natural additives after thermo-oxidative and UV aging. According to the results, it may be concluded that all samples have undergone post–curing due to the influence of elevated temperature and UV radiation. The slight increase of ν_e_ in the case of vulcanizates with addition of freeze-dried extracts compared to the reference system may be a result of antioxidant character of natural substances, which is provided by the presence of phenol compounds. Phenol and its derivatives may act as reducing agents (free radical terminators), metal chelators and singlet oxygen quenchers [108].

According to the presented results, the spatial network of vulcanizates containing antioxidant additives, depended on the solution from which added substances were freeze-dried. Samples with the addition of extracts obtained from water–methanol solutions are characterized by the highest values of ν_e_, while extracts from pure water solution quite opposite. Cross–linking density for NR-CN samples varies from 0.93 × 10^5^ mol/cm^3^ (NR-CN-W_Therm_) to 1.75 × 10^5^ mol/cm^3^ (NR-CN-W/M_UV_), while for NR-MP samples from 1.16 × 10^5^ mol/cm^3^ (NR-MP-W_Therm_) to 1.83 × 10^5^ mol/cm^3^ (NR-MP-W_UV_).

By analyzing obtained cross–linking density, it may be stated that the addition of freeze–dried extracts contributed to the decreased impact of aging factors on the properties of rubber–based vulcanizates.

#### 3.2.3. Barrier Properties

The study of the air permeability of a material is one of the most important features to define, which enables the determination of the future application of a given material for practical purposes. Barrier properties of vulcanized mixtures were established by the gas transmission rate (GTR) to define the impact of added freeze–dried extracts on the natural rubber mixtures.

**Figure 9:**
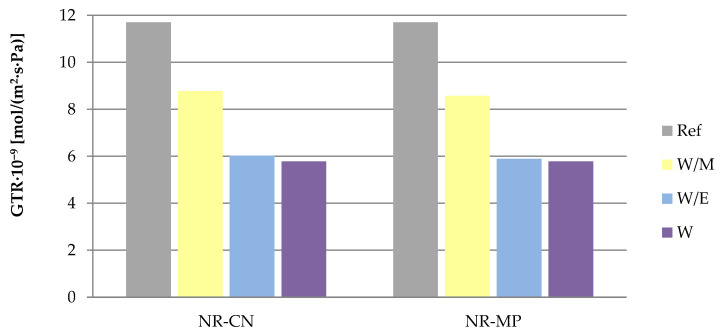
The gas transmission rate (GTR) values of tested vulcanizates.

According to the results (Figure 9), the greatest gas permeability with the GTR parameter equal to 11.7 × 10^−9^ mol/(m^2^·s·Pa) was recorded for the reference system. The addition of natural extracts from water–methanol solution caused a slight decrease of gas permeability values, while the GTR parameter registered in the case of vulcanizates containing water and water–ethanol freeze–dried extracts was reduced almost twice compared to the reference sample. The observed effects were characteristic for both *Mentha piperita* L. and *Urtica dioica* L. extracts. 

The differences in the limitation of gas diffusion is probably related to the obtained cross–linking density of vulcanizates.

#### 3.2.4. Mechanical Properties

**Figure 10:**
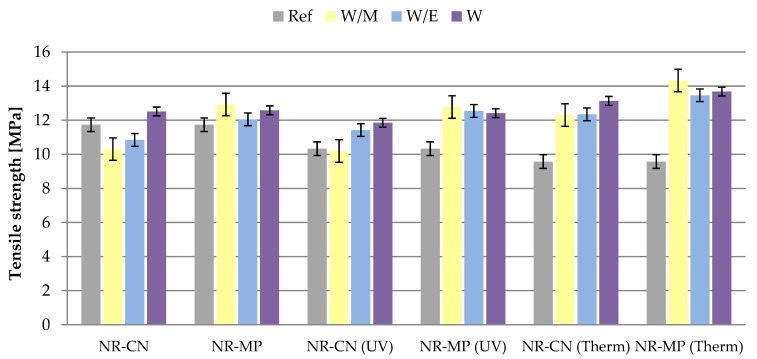
Results of tensile strength measurements of unaged samples, after ultraviolet (UV) and thermo-oxidative (Therm) aging.

**Figure 11:**
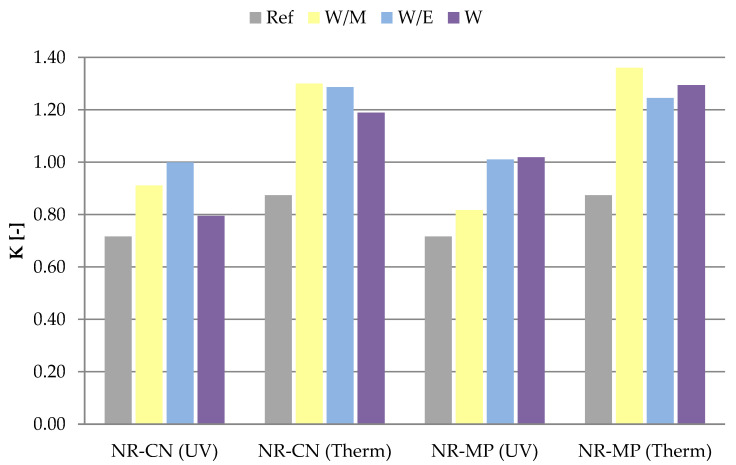
Aging factor (K) of samples after ultraviolet (UV) and thermo-oxidative (Therm) aging.

In practical terms, during the exploitation of articles/products made of polymer vulcanizates, the effect of aging factors also contributes to the change of the mechanical properties of these materials. To determine the resistance of the examined vulcanizates to stress after simulated exposure to thermo-oxidative conditions and UV radiation, the samples were subjected to strength tests, where the tensile strength (TS) and elongation at break (Eb) were analyzed. As an additional parameter, the aging factor (K) was determined as a definition of occurred changes in mechanical properties, where aged samples were compared to the non-aged ones. The results are presented in the table and graphs (Table 7, Figure 10 and Figure 11).

TS values of unaged vulcanizates ranged from 10.3 to 12.9 MPa, where the minimum was recorded for NR-CN-W/M and the maximum for NR-MP-W/M. The reference sample had an intermediate value of 11.7 MPa. The addition of peppermint freeze−dried extract caused a slight increase of TS regardless of the type of the prepared solvent of extraction, while in the case of common nettle the increase was registered only for the additive extracted from pure water. The Eb parameter ranged from 619 to 655% (Table 7), proving the different elasticity of the obtained samples.

By analyzing the tensile strength values for both types of aging, it can be concluded that the aging process improved the mechanical properties of vulcanizates containing natural extracts, while the aging factors reduced the TS of reference system. Tensile strength was higher after aging for the peppermint vulcanizates compared to common nettle, which is related to the antioxidant nature of this plant. This was confirmed by antioxidant activity studies, spectroscopic analyzes and UV-VIS investigation. It was also noticed that the samples containing natural additives presented higher mechanical strength after thermo-oxidative degradation compared to the samples aged by ultraviolet radiation. Deterioration of mechanical strength despite the increased cross–linking of unaged and aged reference sample and vulcanizates after UV aging may be related to the achievement of the so-called critical limit of cross–linking density, beyond which the mechanical properties of vulcanizates begin to deteriorate. According to the elongation at break data, the samples after thermo–oxidative aging showed greater elasticity than the reference samples and vulcanizates after the UV radiation, which correlates with the tensile strength results. 

The aging factor calculated for the samples indicated an improvement of mechanical properties for vulcanizates with the addition of natural extracts after degradation processes. The highest values of the K parameter in the range of 1.25–1.36 were obtained for vulcanizates containing peppermint freeze-dried extracts after the influence of elevated temperature. These samples demonstrated the highest strengthening effect among all samples. The significant improvement of properties was noticed also for NR-CN (Therm) vulcanizates. A slight increase of mechanical strength was measured for vulcanizates containing peppermint from ethanol–water and pure water solutions after UV radiation (K ca. 1.00). All samples with the addition of freeze–dried extracts characterized by the increased aging factor compared to the reference system. 

Both aging processes caused significant deterioration of mechanical strength in the case of the reference samples. The aging factor for these vulcanizates was 0.72 and 0.87 respectively after UV radiation and thermo–oxidative aging. According to obtained results, it can be concluded that the addition of freeze–dried extracts to natural rubber vulcanizates improved the material’s resistance to external aging factors.

#### 3.2.5. Color Stability

**Figure 12:**
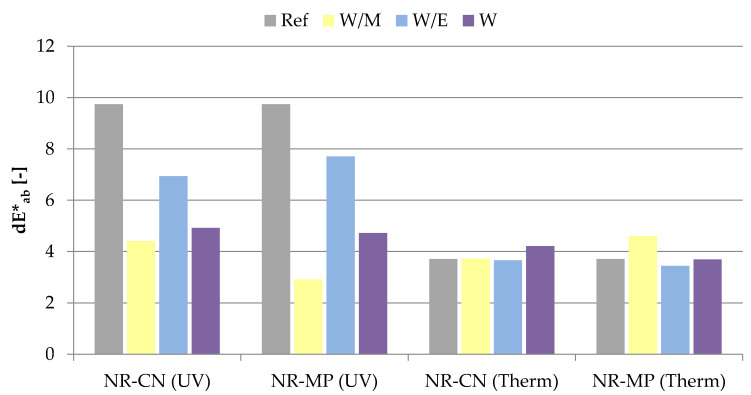
The color change parameter (dE*_ab_) of samples after ultraviolet (UV) and thermo-oxidative (Therm) aging.

The aging processes affect the external appearance of the vulcanizates, which enables in-direct determination of the influence of degrading factors on the condition of aged vulcanizates by measuring the color change (dE*_ab_) of their surface. The results obtained during the color change test are presented in the graph (Figure 12).

The presence of mainly chlorophyll dyes and a smaller amount of carotenoids identified in the UV–VIS study of extracts contributed to the slightly green color of the vulcanized mixtures containing natural additives compared to the unfilled system.

Ultraviolet aging caused the greatest color change in the case of the reference sample, reaching a value of ca. 9.7, while vulcanizates with extracts from water–ethanol solutions were noticed as 6.9 for NR-CN and 7.7 for NR-MP. The color change of the rest of the samples subjected to UV aging did not exceed 5, and the lowest value of 2.9 was recorded for vulcanizates containing *Mentha piperita* L. freeze–dreid extract from water–methanol solution. The reduced dE*_ab_ values observed for filled vulcanizates are related to the presence of antioxidant secondary metabolites in extracts such as flavonoids and phenolic acids. These compounds can react with free radicals in various ways including hydrogen atom and single electron transfers or transition metal chelation [109]. The dE*_ab_ parameter was significantly lower in the case of thermo-oxidative aging reaching values from 3.6 to 4.6 for all samples. According to the results, the addition of freeze–dried extracts to natural rubber mixtures caused a significant decrease of color change in the case of ultraviolet aging. 

## 4. Conclusions

The results of various types of spectroscopy analyses (UV–VIS, FTIR, NIR) provided information about the main building materials and compounds contained in studied freeze-dried extracts of selected plants. According to these data, characteristic plants dyes: chlorophyll “a” and “b” were demonstrated. Moreover, various compounds of secondary metabolites such as polyphenol and its derivatives were found in both *Mentha piperita* L. and *Urtica dioica* L. The antioxidant character of these materials was proved by the studies of total phenolic content (TPC) and antioxidant activity by methods of DPPH and ABTS. Besides, thermal decomposition of prepared bio–additives presented as DTG and TG curves, showed slight differences between peppermint and common nettle freeze–dried extracts, which is related to the plant’s composition and type of solvents used for the extraction process.

The addition of natural freeze-dried extracts contributed to the extension of optimal vulcanization time compared to the reference sample, which might be the effect of the organic phase introduced to the natural rubber mixtures. This could also have an impact on the measurement of maximum and minimum torque. The highest values of increase in torque (ΔM) were calculated for the reference sample. These results correlated with the cross–linking density (ν_e_), which was greater for the reference system both unaged and aged. Phenol compounds present in prepared additives might have acted like reducing agents, metal chelators and singlet oxygen quenchers leading to the decrease of ν_e_ parameter of vulcanizates containing natural additives compared to the reference sample.

Vulcanizates containing freeze–dried extracts improved the barrier properties by reducing the gas transmission rate almost twice compared to the reference sample. Natural additives had a favorable impact on the mechanical strength of prepared vulcanizates especially after thermo-oxidative degradation. According to the results of tensile strength and elongation at break, freeze-dried extracts had anti–aging impact and ensured the additional resistance to external aging factors. This was confirmed by the color change studies, where vulcanizates containing bio–additives were characterized by a lower change of color than reference samples after ultraviolet and thermo-oxidative degradation.

Carried out studies were another attempt to apply the natural materials from a renewable source for the improvement of elastomer vulcanizates properties. According to obtained results the antioxidant activity of selected plants freeze–dried extracts had significant effect on the properties of prepared vulcanizates. *Mentha piperita* L. and *Urtica dioica* L. are natural antioxidants, which caused increased aging resistance and improved mechanical strength of natural rubber–based vulcanizates, thus they can be used as bio–additives for elastomer mixtures. 

## Figures and Tables

**Table 1:** Formulations of rubber mixtures.

Sample Name	Extract	NR	Stearin	ZnO	MBT	Sulfur
	(phr ^1^)					
Reference Sample (Ref)	0	100	1	5	2	2
**NR-CN-W/M ^2^**	5.0	100	1	5	2	2
**NR-CN-W/E ^3^**	5.0	100	1	5	2	2
**NR-CN-W ^4^**	5.0	100	1	5	2	2
**NR-MP-W/M ^5^**	5.0	100	1	5	2	2
**NR-MP-W/E ^6^**	5.0	100	1	5	2	2
**NR-MP-W ^7^**	5.0	100	1	5	2	2

^1^ phr–parts per hundred parts of rubber; ^2^ NR-CN-W/M—vulcanizates with addition of freeze–dried extract of common nettle from water–methanol solution; ^3^ NR-CN-W/E—vulcanizates with addition of freeze–dried extract of common nettle from water–ethanol solution; ^4^ NR-CN-W—vulcanizates with addition of freeze–dried extract of common nettle from water solution; ^5^ NR-MP-W/M—vulcanizates with addition of freeze–dried extract of peppermint from water—methanol solution; ^6^ NR-MP-W/E—vulcanizates with addition of freeze–dried extract of peppermint from water–ethanol solution; ^7^ NR-MP-W—vulcanizates with addition of freeze–dried extract of peppermint from water solution.

**Table 2:** Characteristic functional groups registered by FTIR analysis of freeze-dried extracts. Adapted from ref. [80].

Peak Assignments and Type of Vibration	Wave Number [cm^−1^]
*v* (O–H) phenols & alcohols, –C=O_*w*_ (overtone) and *v* (=C–H_*vw*_)	3600–3000
*v_s_* (C–H) aliphatic and *v*_*as*_ (–C–H_*m*_, –CH_3_, –CH_2_)	2970–2800
*v* (C=O)	1730–1690
*v* (C=C) in aromatic rings	1680–1550
*v*_*vw*_ (–C=C–, cis-) and *d* (–OH)	1675–1648
*v* (C=C) aryl, *d*_*vw*_(–CH_2_) and (–CH_3_) bending (scissoring) or *v*_*vw*_ (–C–H) bending (rocking)	1600–1500
*v* (C–C) aliphatic	1500
*sv* (C=C) aromatic	1441
(O–H) bending	1390–1310
*v* (–C–H, –CH_3_)	1372–1337
*v*_*m*_ (–C–O) or *d*_*m*_ (–CH_2_–), *v*_*w*,*m*,*vw*_ (–C–H, –CH_3_)	1285/1244
*v* (–C–O), *v* (–C–H), *v* (–O–H)	1056
*v*_*m*,*vw*_ (–C–O),	1044/1023
*v* (C–O), (C–C)	1020–1030
*v* (O–C–O)	1090–1020
*v*_*m*_ (–C–O)	1091

Abbreviations: *v*—stretching vibrations; *d*—deformation vibrations; *s*—symmetric; *as*—asymmetric; *st*—strong; *w*—weak; *vw*—very weak; *m*—medium; *sv*—skeletal vibration; *a*—axial.

**Table 3:** Characteristic functional groups registered by NIR analysis. Adapted from refs. [81,87,88,89].

Peak Assignments and Type of Vibration	Wave Number [cm^−1^]
*s* (–CH_n_)	4034–4040
*s* (–C–H)	4375–4408
*s* (–O–H)	4531–4890
*s* (–O–H)	5093–5233
*s* (–C–H) first overtone	5551–5928
*s* (–O–H)	6670–7068
*s* (–C–H) second overtone	8093–8550

Abbreviations: *s*—stretching.

**Table 4:** Percantage mass losses (Δm) of samples in various temperature ranges from 25 to 600 °C and residue after thermal decomposition at 600 °C (R_600_).

Sample	Δm_25–150 °C_ (%)	Δm_150–350 °C_ (%)	Δm_350–600 °C_ (%)	R_600_ (%)
CN-W/M	15.4	34.9	10.0	39.7
CN-W/E	13.8	33.4	12.9	39.9
CN-W	13.0	35.0	13.0	39.0
MP-W/M	7.1	37.2	13.9	41.8
MP-W/E	13.0	35.5	13.5	38.0
MP-W	13.0	35.3	13.3	38.4

**Table 5:** Total phenolic content and antioxidant activity of *Mentha piperita* L. and *Urtica dioica* L. leaf extracts. The results are expressed as mean ± SD.

Sample	TPC (µg_GAE_/mL)	Antioxidant Activity (mg_TE_/mL)	
		ABTS	DPPH
MP-W/M	6206.2 ± 41.8 ^a^	11.75 ± 0.06 ^a^	9.12 ± 0.23 ^a^
MP-W/E	5208.2 ± 276.9 ^b^	9.67 ± 0.05 ^b^	8.89 ± 0.94 ^a,b^
MP-W	540.5 ± 15.7 ^e^	1.45 ± 0.03 ^e^	2.30 ± 0.04 ^c^
CN-W/M	1282.4 ± 54.9 ^d^	1.85 ± 0.01 ^d^	2.25 ± 0.13 ^c^
CN-W/E	2972.5 ± 115.0 ^c^	5.47 ± 0.02 ^c^	8.53 ± 0.06 ^b^
CN-W	61.2 ± 5.2 ^f^	0.09 ± 0.01 ^f^	0.20 ± 0.02 ^d^

^a,b,c,d,e,f^—statistically significant differences between samples (*p* < 0.05).

**Table 6:** Results of the cross–linking density (ν_e_) before degradation process (Unaged) and after ultraviolet (UV) and thermo-oxidative (Therm) aging.

Sample	ν_e_ × 10^5^ (mol/cm^3^)		
	Unaged	UV	Therm
Ref	1.63 ± 0.01	1.98 ± 0.03	1.74 ± 0.02
NR-CN-W/M	1.23 ± 0.04	1.75 ± 0.04	1.36 ± 0.03
NR-CN-W/E	1.19 ± 0.04	1.47 ± 0.03	1.33 ± 0.03
NR-CN-W	0.93 ± 0.03	1.49 ± 0.02	1.26 ± 0.01
NR-MP-W/M	1.60 ± 0.05	1.83 ± 0.05	1.63 ± 0.04
NR-MP-W/E	1.24 ± 0.04	1.49 ± 0.05	1.37 ± 0.03
NR-MP-W	1.16 ± 0.04	1.36 ± 0.04	1.33 ± 0.02

**Table 7:** Elongation at break before (Eb) and after ultraviolet (Eb_UV_) and thermo–oxidative (Eb_Therm_) aging.

Sample	Eb (%)	Eb_UV_ (%)	Eb_Therm_ (%)
Ref	649.15 ± 4.48	527.74 ± 6.41	695.57 ± 8.31
NR-CN-W/M	646.47 ± 5.22	595.44 ± 4.14	748.33 ± 7.91
NR-CN-W/E	655.38 ± 5.23	621.50 ± 4.22	740.60 ± 6.34
NR-CN-W	645.40 ± 6.22	541.56 ± 2.49	731.39 ± 2.58
NR-MP-W/M	639.47 ± 7.18	528.49 ± 5.65	770.43 ± 9.98
NR-MP-W/E	668.26 ± 6.89	648.95 ± 5.37	744.90 ± 7.68
NR-MP-W	619.08 ± 5.81	639.61 ± 4.47	736.87 ± 5.86

## Data Availability

Data sharing not applicable.
